# Effects of hormonal contraceptives on sleep - A possible treatment for insomnia in premenopausal women

**DOI:** 10.5935/1984-0063.20180025

**Published:** 2018

**Authors:** Andreia Gomes Bezerra, Monica Levy Andersen, Gabriel Natan Pires, Sergio Tufik, Helena Hachul

**Affiliations:** 1 UNIFESP, Departamento de Psicobiologia - São Paulo - SP - Brasil.; 2 Santa Casa de São Paulo School of Medical Sciences, Departamento de Ciências Fisiológicas - São Paulo - SP - Brasil.

**Keywords:** Sleep, Insomnia, Contraceptives, Estrogen, Progestagen, GABA

## Abstract

Due to the changes that took place since the 1970s, women have achieved important socioeconomic positions. Many tasks, including domestic and familiar ones, continue to be under women’s responsibility, which leads to an overload work. Additionally, the female organism has its peculiarities due to hormonal changes. Adding all these factors up, women seem to be more vulnerable to stressing factors and consequently, might be prone to present several health problems. Within this scenario, one can point out insomnia as a highly prevalent disease among women, directly affecting performance and quality of life. Progesterone has an important effect over sleep, acting both as a hypnogenic and as a respiratory stimulant. Hormonal contraceptives are largely recognized among the modern society women; however, little is known about the effects of these drugs on sleep. This proposal hypothesizes that the use of hormonal contraceptives, mainly those based on progestagens could be a new therapeutic element for the treatment of insomnia and one more tool to be used to improve women´s sleep pattern and quality of life.

## INTRODUCTION

### Women, social context and health

Currently, women perform an important socioeconomic role, which has been progressively conquered. Due to the changes that took place since the 1970s, women now have the freedom to look for education, finding a job and be financially independent. However, the equality among genders is not yet totally established^1^, as their increased importance and participation in society was not accompanied by decrease on long-standing classical women-related activities, such as household responsibilities and maternal tasks. This partial alteration in women’s lifestyle may bring an overload and markedly increases the vulnerability to stressing factors and, consequently, to health risks and compromised well-being^2^^,^^3^.

In conjunction to this social scenario, the female organism seems to be more vulnerable to stressing factors by nature. This predisposition is well explained by the hormonal changes that occur during the menstrual cycle. The premenstrual or luteal phase is frequently associated with mood alterations, being negativity and irritability very commonly observed^4^. When these symptoms affect social life, relationships and work, it may be a case of premenstrual syndrome. During this phase, women seem more prone to anxiety and depression disorders, as well as increased sleepiness and insomnia^5^.

In this sense, it might be noticed that two concurrent factors, among others, contribute to women’s high predisposition to stress^2^^,^^3^: 1. The lifestyle modern women are subjected to, which leads to overlap of several social, work and domestic activities, and 2. Women’s hormonal background, which explains related factors, such as mood oscillations and higher likelihood to anxiety and depression disorders. Despite parallel and independent, these two factors share an important feature: both are closely related to women’s sleep pattern.

### Women’s sleep

Sleep is an important aspect of the female physiology, as it appears to be directly influenced by hormonal variation. This influence can be observed both in short term, concomitantly with menstrual cycle, as well as in long term, ontogenetically from early life until the postmenopausal period. Regarding the sleep across the menstrual cycle, evidences suggest that sleep is mostly disturbed during the mid-luteal phase, when steroidal hormones levels start to decline^6^. During this phase, women tend to experience an increased number of awakenings and arousals during sleep, if compared with the follicular phase^7^^-^^9^. These findings attest the impact of hormonal oscillations on the onset of sleep complaints. The same impact might be seem across the lifespan, as shifts on hormonal patters observed during puberty and menopausal transition are associated with increased prevalence of insomnia^6^.

**Figure 1:**
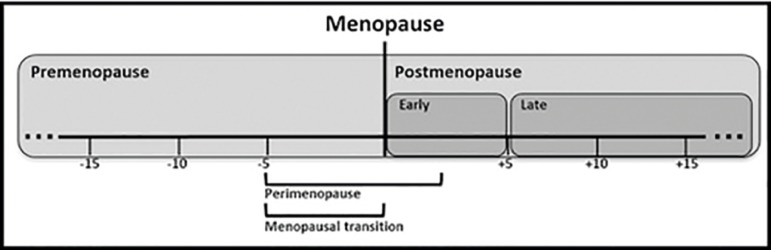
Women’s life-span and menopause-related events. Premenopause representes the entire period before the menopause. Adapted from Hachul et al (78).

Considering premenopausal women (i.e. women on their fertile age range, from the menarche until the menopausal transition - not to be confused with peri or postmenopause - Figure 1), they present a unique pattern of sleep disorder, reasonably different from what is observed in men and closely related to a hormonal background^6^^,^^10^^-^^14^. Subjective complaints of disrupted and insufficient sleep, poorer sleep quality, difficulties falling sleep, frequent night awakenings, longer awake periods after sleep onset and recurrent nightmares are all more frequently reported by women in comparison to men^13^^,^^15^^-^^18^. These complaints might be associated with sleep disorders, such as insomnia and obstructive sleep apnea. Gender specificities play an important role in these sleep complaints, as it seems to be increased in women with irregular menstrual cycles^12^, during the menstruation^19^ and in those with severe premenstrual syndrome^9^.

Among all the sleep-related complaints reported by premenopausal women, the increased prevalence of insomnia is probably the most remarkable and clinically relevant sleep characteristic^20^. The prevalence of insomnia is somewhat difficult to achieve due to methodological caveats, such as the definition of insomnia, the diagnostic criteria, sampling biases among studies and source population^20^. Even so, it is estimated that about 12-40% percent of all women reports insomnia-compatible symptoms^21^^-^^29^. In all cases, women are twice as likely to experience insomnia throughout their lifespan compared to men^30^, and the female to male prevalence ratio of insomnia is approximately 1.5/1^31^. Taking together, these studies indicate a strong relationship between hormonal variation observed in women and the higher prevalence of insomnia.

### Sleep and female steroidal hormones

As aforementioned, hormonal oscillation seem to underlie all major sleep complaints in women, as observed across the menstrual cycle and in association with puberty, pregnancy and menopausal transition^6^. As an example, it has been suggested that the increase on the prevalence of sleep complaints and disorders in women during post menopause occurs due to the decrease of the plasmatic levels of steroidal hormones^32^. Despite several studies have suggested a direct relationship between steroidal hormones and sleep, the mechanisms behind these sleep changes are still poorly understood. Regarding the role of hormones on sleep, it is suggested that the two major female sexual hormones classes are involved: estrogens and progestagens.

It is argued that progestogens have an important hypnotic effect. According to the results of a double-blind placebo controlled crossover trial, progestagen administration in men leads to a significant increase on slow-wave sleep and decrease on the slow wave frequency spectral power during non-REM sleep, which resembles the EEG profile induced by gabaergic drugs^33^. It leads to reduction on sleep latency, as well as some positive effects on sleep disorders such as periodic limb movements disorder, bruxism and obstructive sleep apnea syndrome (for a review, see Andersen et al.^34^). Progesterone is also a potent respiratory stimulant, being related to an increased dilation of the superior airway as well^34^. Therefore, the decrease on progestagen levels explains the development of sleep obstructive apnea syndrome on postmenopausal women^35^.

Studies conducted in animal models have provided information regarding the potential mechanism of the hypnotic effects of progestagens. It has been verified that progesterone is capable of altering the sleep pattern as an agonist of the GABA-A receptors^36^. This effect was reversed when the animals received an antagonist of the GABA-A receptor, confirming the relationship between the progesterone and gabaergic receptors^37^. It is very likely that progesterone does not act directly on GABA-A receptors though, but rather involves the activity of a metabolite named allopregnanolone (5α-pregnane-3α-ol-20-one). This neurosteroid is the final responsible for the GABAergic agonism^38^, being also related to other GABA-related effects, such as anxiolysis^34^^,^^38^.

Estrogens also have an important effect on the sleep pattern, despite slightly less well understood than the effects of progestagens. Evidences coming from estrogen replacement therapy suggests a hypnotic effect, since it ameliorates most of the sleep-related complaints of perimenopausal women^6^. Additionally, data on rodents suggest that estrogen seems to have an important effect on the consolidation of the sleep-wake cycle, as ovariectomized female rats treated with estrogen display a better balance of sleep when compared with control-ovariectomized females. In this case, estrogen promoted both REM and non-REM sleep during the light phase (the typical sleep period in rodents) and reduced it during the dark phase (typical wakefulness period in rodents)^39^^,^^40^. The mechanisms underlying hypnotic effects of estrogen probably involves signaling via E2 receptors on areas such as the ventrolateral preoptic area, involved on sleep onset and maintenance, and the lateral hypothalamus, where hypocretinergic neurons are located^6^.

Regardless of the isolated effects of each of these hormones, the actual effects of sexual hormones on sleep, including its hypnogenic potential, its effects on sleep architecture and its benefits over sleep complaints seem to be a combination of both estrogen and progestagens. It not clear how much each of these hormones contributes to these sleep modulations.

### Insomnia and pharmacological treatment

According to the DSM-V, persistent insomnia is defined as a difficulty to initiate or maintain sleep, with frequent awakenings or difficulty in reinitiating sleep. It must be present for at least three months, occurring at least three nights a week, with diurnal effects^41^. Its importance has been highlighted due to its diurnal repercussions which comprises 10% of the population^42^, with loss of concentration, mood alterations and tiredness^43^. Concurrently to persistent insomnia, DSM-V also defines episodic insomnia, similar to the previous but with duration of one to three months, and recurrent insomnia, when two or more episodes are diagnosed in one year^41^.

Considering the increased prevalence, insomnia is an important matter from a public health perspective, due to its high social and economic impact^31^^,^^43^. This condition represents a significant burden to public health, as it leads to increased demand for medical and psychiatric care^43^^,^^44^. It also represents a problem for employment relationships, since insomnia has been related to reduced productivity, decreased work efficiency and absenteeism^43^^,^^44^. It is estimated that the overall costs related to insomnia, both related to direct and indirect expenses, are between U$92.5 and U$107.5 billion per year in the United States^31^.

Additionally, to its public health aspects, insomnia is also a significant problem at individual level, since it is associated with several psychiatric and medical consequences. It has been associated, either in short or long term, with depression, anxiety, substance abuse, cognitive impairment, metabolic disorders (diabetes, dyslipidemia and obesity) and cardiovascular disease (for review, check^45^). As insomnia is markedly more prevalent among women than in men (as mentioned on the *women’s sleep* section), all these co-morbidities impact women on a bigger proportion.

There is an intense search for therapies to reduce the complaints of insomnia and all its related consequences, especially among women. Presently, non-benzodiazepine hypnotics, known as Z-drugs, has been largely employed for the treatment of insomnia, among which one can highlight zolpidem^46^. This drug acts as a selective agonist of the alpha-1 subunit of the GABA-A receptor, the main receptor responsible for the inhibition of neurotransmission in the central nervous system. It is highly prescribed in the whole world due its short term effectiveness, as well as for its safety, very high when compared with the entire class of the benzodiazepines^47^.

Some studies have demonstrated the efficacy of zolpidem in improving the quality of sleep in patients with chronic insomnia^48^^-^^50^. However, despite its general good efficacy and safety, some reports of side effects and reduced therapeutic potential has been published, encompassing tolerance, rebound insomnia, residual effects, impairments on motor performance and memory deficits^51^^,^^52^. Reports have demonstrated an abuse potential for zolpidem^53^, although lower then with benzodiazepines, as well as some associations with psychosis, amnesia, parasomnias, hallucinations, suicidal ideation and other side-effects^54^^-^^56^.

Considering the significant prevalence and consequences of insomnia and the limitation reported on the continuous use of zolpidem, new pharmacological therapeutic strategies should be sought. This is especially relevant when insomnia is secondary co-morbid to other conditions. In such conditions, drugs acting on the primary condition might be more adequate to treat insomnia. As examples, one may cite the use of antidepressants (e.g.: amitriptyline, trazodone, doxepin and mirtazapine) when insomnia is co-morbid with major depression; atypical antipsychotics (e.g.: olanzapine and quetiapine) when it is secondary to bipolar disorder or psychotic episodes; or anticonvulsants (e.g.: gabapentin, pregabalin and gaboxadol) when it arouses from epilepsy or chronic pain. In these cases, those specific drugs might be more specific to the mechanisms of the underlying cause of insomnia.

This condition is similar to the observed in women, case in which the pathophysiology of insomnia seem to be linked to a hormonal background^57^^,^^58^. Consequently, steroid hormones levels might be seen as a potential mechanism for therapeutic approaches^6^^,^^34^.

## HYPOTHESIS

Based on the aforementioned discussion, we hypothesize that sexual hormones (mainly progestagens) used as contraceptives could have positive effects on the sleep pattern in premenopausal women. Our hypothesis is driven by four main facts: 1. the high incidence of insomnia and sleep complaints among women; 2. the sleep-promoting effects of sexual hormones, 3. the promising results acquired in preclinical research and 4. the benefits of treating insomnia and its comorbidities with a single therapeutic approach.

A few trials about the effects of contraceptives on sleep supports this hypothesis, despite of some inconsistencies on the results. Previous epidemiological studies from our research group have shown that oral contraceptive users display a reduced incidence of snoring and awakenings^12^^,^^59^. Polysomnographic data proves that women who were using hormonal contraceptives demonstrated better sleep efficiency and a smaller sleep apnea and hypopnea indexes when compared with women that did not use hormonal contraceptives in different menstrual cycle phases^59^.

Further studies also show increase in N2 sleep, despite presenting a decrease in non-REM sleep time^60^^,^^61^. Data from hormonal replacement therapy in postmenopausal women also support this hypothesis, being effective in reducing sleep complaints during this period^30^^,^^62^. Combined hormonal therapy is also effective in decreasing the severity of apnea in women from 50 to 59 years of age^63^. Conversely, a recent trial have failed to detect any relevant sleep-promoting effect on the use of estrogen-progestin therapy^64^.

Hormonal contraceptives are available either as a combination of two hormones (progestagen and estrogen) or as a single hormonal component (usually progestogen). They are available in different formats and administrations protocols, including oral contraceptives, intravaginal rings, and transdermal patches, among others. It shall be pointed out that most of the studies on the field either used only one type of hormonal contraceptive (progestagen-only or combined presentations) or did not controlled which hormonal contraceptive the participants were using. This in an important limitation, once there is an enormous variety of synthetic estrogens and progestagen in the market. The lack of a proper control and reporting of the type of contraceptives being used in clinical trials introduces experimental biases and impairs to address adequately its potential therapeutic effects.

Few studies have focused on more specific and unbiased hormonal administration, and those focusing on progestogens have provided promising results. On a recent trial Leeaunkulsathean et al.^65^ showed that both micronized progesterone and dydrogesterone significantly improved sleep quality in peri-postmenopausal women with insomnia. These results are corroborated by several previous studies, all of them reporting positive effects of progestagen therapy on sleep in postmenopausal women^11^^,^^66^^-^^68^. All these studies have focused on polysomnographic outcomes such as sleep efficiency, time spent awake after sleep onset and other objective polysomnographic measures. This shows that the applicability of progestagens over sleep goes further than its effects on sleep apnea and breathing, affecting directly sleep continuity measures.

It is worthy to mention that the contraceptives have a specter of action much bigger than its primary function, being regularly used for indications other than contraception itself. Contraceptives are the current choice therapy for the treatment of polycystic ovaries syndrome^69^ and dysmenorrhea^70^. Other studies indicate that the use of a combined oral contraceptive is also effective for the treatment of facial acne^71^ and other androgynisms, such as seborrhea, alopecia and hirsutism^72^. In the same way, there is a reduction of the risk of ovarian, endometrial and colorectal cancer development, among the users of hormonal contraceptives (for revision, see Schindler^73^). Specifically to progestogens, these drugs present effects not only on contraception, but also on other hormone-related condition and even in cognitive and behavioral functions^74^. Therefore, the current proposal expands the therapeutic potential of contraceptives to insomnia in women; a condition deeply related to sexual hormones just as all the above.

Every secondary use of contraceptives requires a thoughtful investigation about the better hormonal composition, doses, treatment schedule and route of administration. This detailed analysis has never been performed for the potential hypnogenic effects of contraceptives. Most of the trials relating contraceptives and sleep have been composed by convenience samples, or have had sleep measures as secondary outcomes. Such lack of specificity is the possible reason for the inconsistencies on the results disclosed above. It is also the reason why estrogen-based therapies or combined contraceptives cannot be disregarded from its potential sleep promoting effects, even considering that present data points out to progestagen as a more reliable therapeutic option.

In summary, we hypothesize that the use of contraceptives could be taken as a therapeutic alternative to treat insomnia in premenopausal women. A definitive conclusion on the therapeutic use of contraceptives for insomnia depends upon a proper and unbiased analysis of their hypnogenic potential (as disclosed on the next section).

## RESEARCH AGENDA

Considering the complexity of the current hypothesis, one single study would hardly be able to address it on its whole. A double-blind clinical trial on the use of contraceptives among premenopausal women with insomnia would be obviously the best choice to address the present hypothesis. However, a single clinical trial cannot address all the possible variations on the use of contraceptives and its potential effects on sleep and insomnia. In this sense, prior experimental and theoretical approaches are needed as preparatory steps for a proper clinical trial. These previous steps would provide information about which are the best hormonal composition, doses, treatment schedule and route of administration to be tested on a clinical trial. Below a research agenda is proposed, designed in order to address the present hypothesis:

1. *Population-based cross-sectional studies*: Epidemiological transversal data might be useful to dissect the contraceptives being used by a given population and the profile of the women using each different type of contraceptive. A few transversal epidemiological studies have collected data about contraceptive use and sleep (such as the São Paulo Epidemiological Sleep Study - EPISONO^59^), but more detailed population-base data are warranted, especially on what regards the prevalence of use of different types of hormonal contraceptives.
2. *Clinical meta-analysis:* A meta-analysis on the effects of contraceptives on women’s sleep would be a clever way to gather all the data available on the field and to synthesize the current evidence. If there were enough data to draw conclusions, raised from well-designed clinical studies, a meta-analysis would be able to detect them, providing relevant clinical data. As per the literature review done to draw the current hypothesis, it is likely that there is no data for such purpose, mainly due to the heterogeneity among study designs, populations, outcomes and contraceptives tested. In any case, only a properly designed systematic review and meta-analysis on the field would be able to gather the literature, summarize the evidences on a reliable fashion and provide the state-of-the-art about the relationship between contraceptive use and sleep.
3. *Preclinical meta-analysis:* Pre-clinical meta-analysis (or meta-analysis of animal data) is an innovative experimental research tool, usually employed under a translational research context. Despite not as usual as the regular clinical meta-analyses, preclinical meta-analysis have gained attention over the last few years, serving for the purposes of both experimental and clinical researchers. This research method arose due to the need to comprehensively overview the literature on animal studies before any new clinical trial^75^^-^^77^, providing solid background information regarding mechanisms, pharmacology and other related issues. In this sense, a meta-analysis about sexual hormones administration and sleep would not be performed only with the intention to summarize effects, but rather to explore data. It might aim on topics such as the possible effect of different hormonal sources, doses, administrations routes, administrations schedules, etc. Additionally, these pre-clinical meta-analyses could provide insight about the specific mechanisms of action for the hypnogenic effects of sexual hormones. In any case, this meta-analysis should be able to provide information to drive a better clinical trial design.
4. *Clinical trials:* Only after these previous steps, which could be done in parallel, a clinical trial would be feasible. The data arisen from the aforementioned studies would be critical for a proper study design, as it would define which are the best hormonal contraception formulations to be tested, the most feasible doses and the best treatment schedules. In other words, the preliminary steps would define the details on a potential clinical trial. An additional point regards a proper evaluation of the costs and expenses encompassed on designing and running a clinical trial. Due to the limited amount of data, a trial on this field would only be feasible and economically justifiable if preliminary data demonstrates potential positive results. In the case of sustained negative data on these previous steps, mainly on the meta-analyses, it would probably be reflected on negative results on further clinical trials. If the performance of clinical trials are justifiable based on previous results, we recommend not to set a general trial, but rather to first analyze the effects of micronized progesterone, as it has already demonstrated relevant positive effects on the sleep of postmenopausal women. A second clinical trial could then evaluate commercial contraceptives in different formulations, doses and treatment regimens. Another important approach on these trials would be to understand the different possible effects of each of the four generations of progestagens available.

Each of the proposed steps on research agenda presented above has its limitations. For instance, cross-sectional studies cannot stablish causal relationships, meta-analysis are subjected to the limitations, heterogeneity and biases on the original studies, and clinical trial encompasses different challenges on the definition of an appropriate experimental design and sample selection. In any case, as long as the limitations are specific to each proposed step, the outcomes are common point among them all. Thus, we believe that four steps altogether will be able to generate a good estimate on the actual effect of contraceptives on premenopausal women’s sleep, by diluting the effect of possible research biases and aggregating evidences on the field.

## CLINICAL AND PRACTICAL IMPLICATIONS

As mentioned above, insomnia and other sleep-related complaints could be better treated by aiming at its primary cause. It is very likely that insomnia and other sleep-related complaints in women have their roots on sexual hormones, reason why they are markedly more prevalent in women. In some degree, such statement provides a syndromic nature to insomnia, in which both the sleep complaints and other symptoms (mood oscillations, dermatological issues, anxiety) are results of a common cause. Other clinical benefits on the potential use of hormonal contraceptives to treat insomnia on premenopausal women encompasses:

- *The benefits of a single therapy*: Usually, single therapies should be preferred to treat composed conditions, rather than polytherapy, as it might promote a better follow up, dosage control and less side effects.
- *Mechanisms of action:* Contraceptives would be a good attempt to treat insomnia based on its mechanisms of action.
- *Well-accepted among premenopausal women:* The use of contraceptives among premenopausal women is already common and well accepted.
- *Costs:* In general, contraceptives, hypnotics and benzodiazepines are accessible and inexpensive drugs. If contraceptives prove to be an efficient alternative therapy for insomnia in premenopausal women, we would promote equivalent effects to the currently used drugs, with no increase on the overall treatment costs.

## CONCLUSIONS

An important aspect of the modern woman´s life was the introduction of the use of contraceptive methods. These instruments allowed the permanence of woman at the workplace and consequently, a later maternity. From the several existing methods, we highlight the use of hormonal contraceptives, which can be used in different configurations: pills, epidermic adhesives and intravaginal rings.

There are several studies about the use of hormonal therapy and sleep in women during the peri and post-menopausal phases, besides the results obtained by experimental models, suggesting that there are steroidal hormone effects upon sleep induction. However, there are few studies evaluating the effects of the hormonal contraceptives on sleep parameters in women during reproductive stages. Non-benzodiazepine hypnogenics are the newest generation for the improvement of sleep disturbances, especially in women. On the other hand, hormonal contraceptives are commonly used premenopausal women and its effects might be equivalent to those of zolpidem and drugs alike.

The investigation based on this hypothesis could provide one more therapeutic element at the physician´s disposal when prescribing a determined contraceptive to the patients, allowing for the adjustment of indication to their individual complaints and for the treatment of possible sleep alterations. The use of a hormonal contraceptive with only one isolated progestagen, a sleep inducing hormone, might modify the quality of sleep of women with insomnia.
